# Novel fiber-tip micro flowmeter based on optofluidic microcavity filled with silver nanoparticles solutions

**DOI:** 10.1515/nanoph-2022-0505

**Published:** 2022-10-24

**Authors:** Jinjian Li, Jian Qu, Yi Liu, Yan Li, Shiliang Qu

**Affiliations:** Harbin Institute of Technology, Harbin, Heilongjiang, China; Harbin Institute of Technology Weihai, Weihai, Shandong, China; Harbin Institute of Technology Weihai, 2 West Wenhua Road, Weihai 264209, China; Harbin Institute of Technology, 92 xidazhi street, Harbin 150001, China

**Keywords:** flowmeter, optofluidic microcavity, silver nanoparticles

## Abstract

A novel fiber-tip micro flowmeter based on optofluidic microcavity filled with silver nanoparticles solutions (SNS) is proposed. CW fiber laser was used to heat SNS that can emit heat obviously due to the excellent optic-thermo effect. The heat generated by the silver nanoparticles would be taken away as the microfluidic flows over the fiber microcavity until thermal balance is established under different velocity. The effective refractive index (RI) of the SNS changed followed by temperature of the thermal balance. The dips of the Fabry–Perot interference spectrum shift and the flow velocity can be demodulated. Moreover, the sensor can measure the flow rate with a high sensitivity due to the superior thermal conductivity and specific heat capacity of sidewalls. The max flow rate sensitivity can reach 1.5 nm/(μL/s) in the large range of 0–5 μL/s with a detection limitation (DL) of 0.08 μL/s. The Micron scale probe-type flowmeter has strong robustness and can be used to measure flow rate in tiny space. The heating medium also has an excellent biological compatibility and is not contact with the fluidics directly. As such, we believe that the proposed fiber-tip micro flowmeter has great application potentials in haematology, oil prospecting, ocean dynamics and drug research.

## 1 Introduction

Micro total analysis system (μ-TAS) has been focused immensely in recent years. Flow rate, a vital parameter in μ-TAS, has also attracted much interests due to its application potentials in chemical analysis, biological applications, clinicopathologic analysis and drug development [1–4]. Micro flow rate sensor can realize the local and real time microfluidics flow rate monitoring especially in some fields replying on precise flow controlling with high sensitivity and wide dynamic sensing range. Traditional micro flow rate monitoring method as micro electro-mechanical systems (MEMS), mainly relies on electrical and mechanical detection technology, utilizing the thermal transfer [5], electrical admittance [6], cantilever deflection [7] and so on.

MEMS provides a promising approach with high integration and impressive performance, However, it is greatly restricted in most biological and chemical laboratories because of high cost and complicated fabrication process. Particle image velocimetry (PIV) measures the movement speed of water quality points by measuring the displacement of liquid quality points within a known time interval [8, 9]. The electromagnetic flowmeter [10–12] based on liquid crystal displays and polarized filters can achieve the flow velocity measuring, but there are still shortcomings such as expensive equipment, small measurement range and low accuracy, which are used for measuring large flow rate. Thus, a fast, accurate and real-time micro flow rate measuring method is needed urgently.

Optical fiber flow rate sensor has developed rapidly due to its unique characteristics such as high sensitivity, compact size, anti-electromagnetic interference and easy integration [13–15], which has been used widely in recent years [16–19]. Most optic fiber flow rate sensors mainly rely on laser Doppler velocimeter (LDV) [20, 21], “hot wire” [22, 23], and some special structure [15, 24, 25]. The laser used in LDV is easily absorbed by living cells, tiny particles and suspended particulates, as a result, the intensity of pump laser decreases dramatically, which has great limitations in flow rate measuring. The special optic fiber structure such as peanut structure [24], bowknot type [15], and optical fiber turbine [25] are mainly utilizing the changes of bending [24], pressure [25] and other intermediate values from the flow rate to effect parameters of the optic interferometer to realize the flow rate monitoring. Traditional “hot wire” fiber sensor is mainly based on Fabry–Perot interferometer (FPI) [26], Mach–Zehnder interferometer (MZI), Michelson interferometer [27], FBG [28–30] and some special optical fibers [31–33]. The sensor probe is mainly based on the heat transfer from sensors to the surrounding environment. The more internal heat of the radiation would be taken away when the microfluidic flow rate increased and the more the spectrum shifts. Generally, the sensitivity of the “hot-wire” flowmeter seriously depends on the interaction length between the light and microfluidic and the temperature response of the fiber device. Several new techniques have been used to improve the “hot wire” sensor performance in recent years such as the film coated optical fiber [34, 35], wrapping structure [36] and high-attenuation fiber [37]. As mentioned above, metal coated optical fiber and high-attenuation fiber of the “hot wire” sensor are all applied to improve the heat production to create a higher temperature difference between the sensor element and the environment. However, in terms of some fluid measuring, the current “hot wire” flow rate sensing probe is heated and contact to the fluid samples measured directly just like a thermocouple. These may damage the fluid characteristics of the sample under test and have significant limitations in biological fluid flow rate measurement. Thus, an ultra-compact optical fiber flow rate sensor is required.

**Figure 1:**
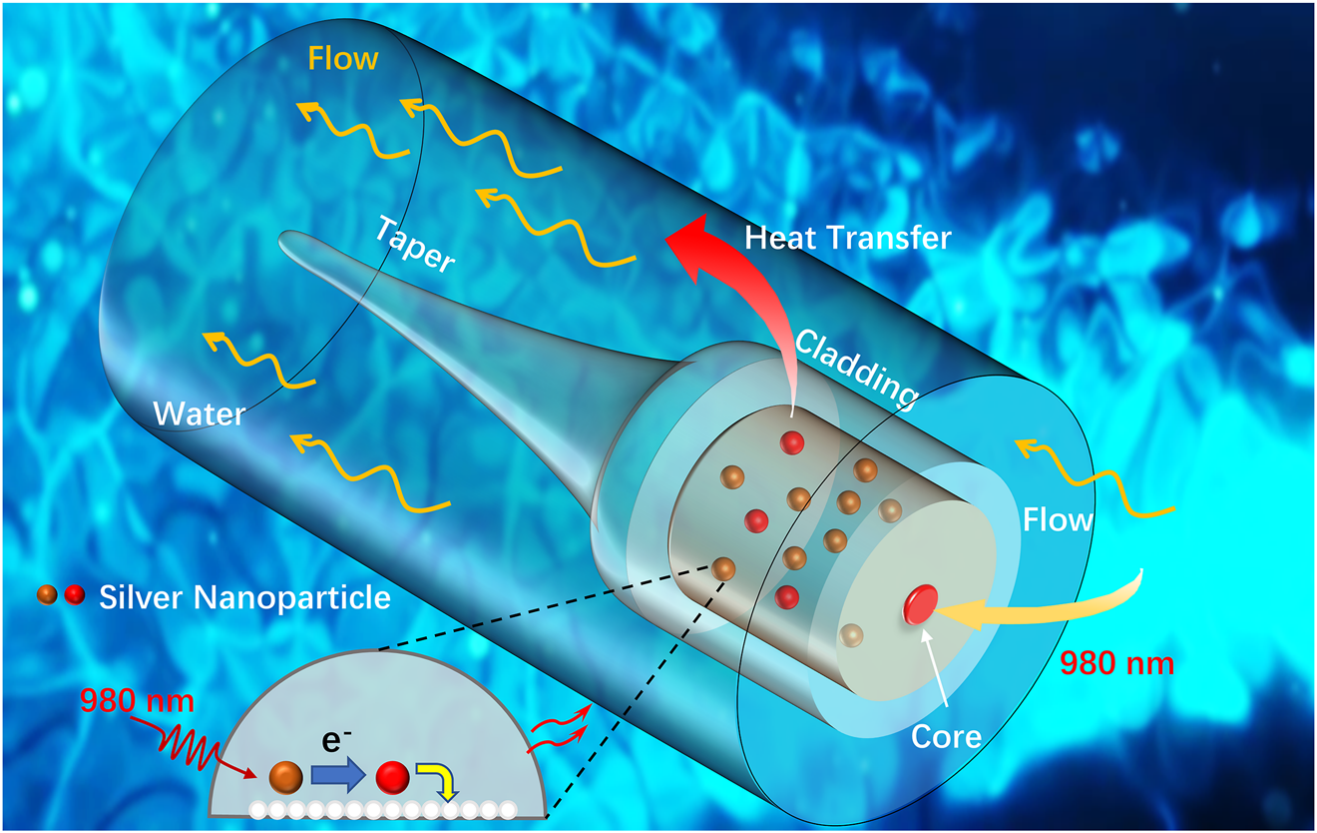
Schematic of the sensor probe. Schematic diagram of all-fiber micro flowmeter based on optofluidic microcavity filled with silver nanoparticles.

In this work, we demonstrate a novel fiber-tip micro flowmeter based on optofluidic microcavity filled with silver nanoparticles solutions (SNS). The structure of the flowmeter as shown in Figure 1 was optimized and simulated using finite element analysis (FEA). As the fluidics flows over the sensor probe of flowmeter, the heat generated by the silver nanoparticles solutions (SNS) was taken away by the fluidics and the temperature decreased, and the effective refractive index (RI) of the silver nanoparticles in the cavity changes causing Fabry–Perot interference dips shifting. Pump laser source is used to heat the SNS in the microcavity until the temperature reaches a dynamic thermal balance with the microfluidics under different flow rate in the channel. The flowmeter can measure the flow rate of various liquids with a high sensitivity due to the excellent thermal optic properties. The max flow velocity sensitivity can reach 1.5 nm/(μL·s) in the large range of 0–5 μL/s with a detection limitation (DL) of 0.08 μL/s. Besides, the novel sensor has a low temperature cross and RI cross. Providing comprehensive insights into micro flow rate changes in tiny space, the micron scale probe-type flowmeter also has strong robustness. The medium being heated is not contact directly with the fluidics under test, and has an excellent biological compatibility. As such, the novel flowmeter provides a new method and opportunity for fluidics flow velocity measuring in μ-TAS.

## 2 Simulation of flowmeter probe

### 2.1 Working principle

Figure 1 shows the principle and simulation model of sensing probe of fiber flowmeter. Some electrons of silver nanoparticles are converted into thermo electrons as 980 nm laser emitted. The non-thermo electrons changes to thermo electron via electron–electron scattering [38, 39], which takes about 500 fs. And then the temperature of thermo electrons increases. Thermalized electrons will further transfer heat to silver ions, and the heat was transferred into the solutions, that is, the heat generation process of SNS in the fiber microcavity. The heat is taken away and the temperature decreases as the microfluidic flows. Subsequently, a dynamic thermal balance would be established. The governing equation for the flowmeter model is the heat equation for conductive and convective heat transfer:

(1) $$\rho C_{p}\nu \cdot \nabla T+\nabla \cdot \left(-k\nabla T\right)=Q$$

where *C*_*p*_ denotes the specific heat capacity, *T* is the temperature, *k* is the thermal conductivity, *ρ* is the density, *ν* is the flow velocity vector, and *Q* is a sink or source term. It is worth noting that, distribution of flow field inside the pipe and the local flow field in the channel may be affected as the fiber probe is inserted into the channel.

**Figure 2:**
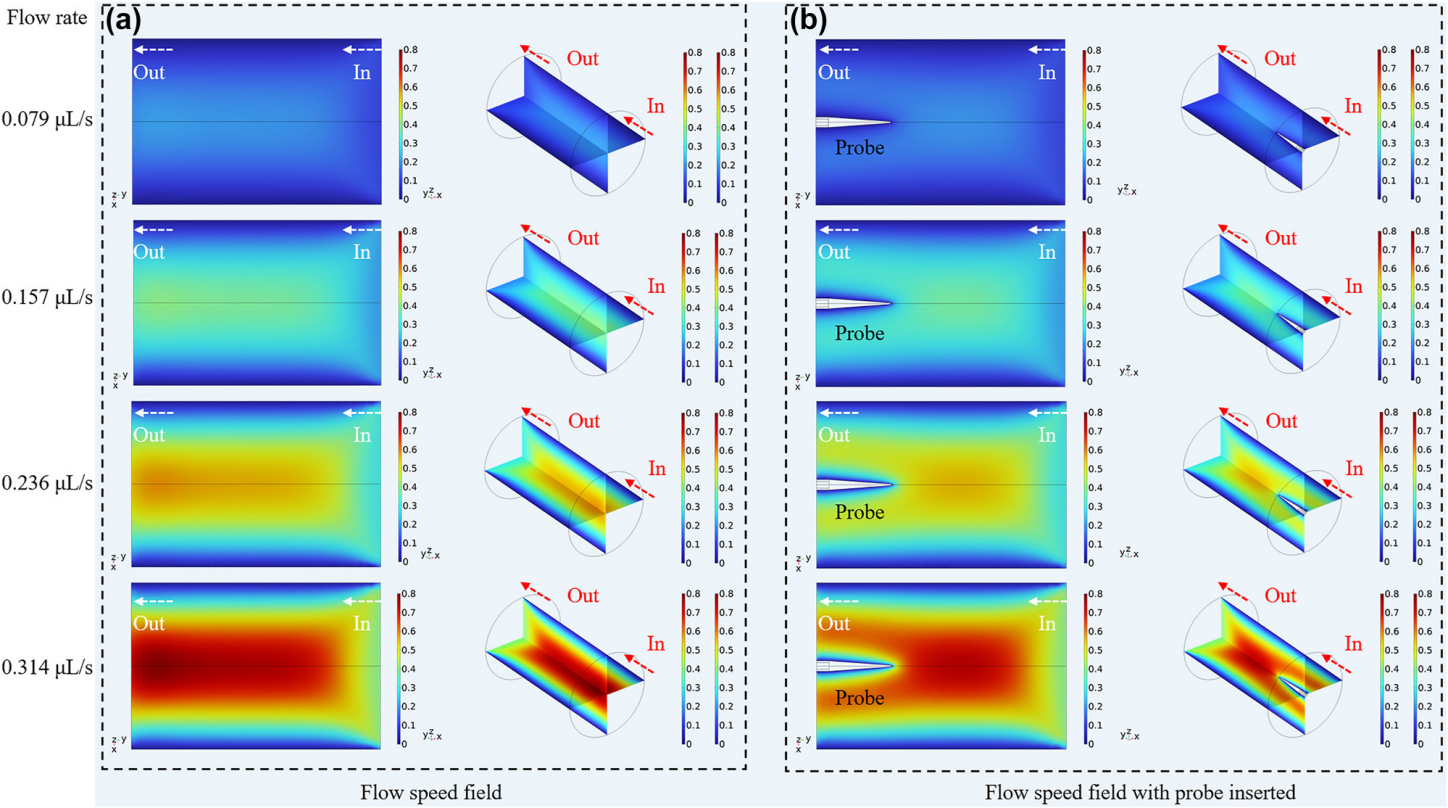
Distribution of speed field in channel under different flow speed: (a) without probe inserted, (b) with probe inserted.

To further study the influence of the flowmeter on the local flow speed in the channel, the local flow field under different flow rate is simulated by FEA as shown in the Figure 2. The flow rate controlled by the syringe pump is a mean value and the difference among the flow speed at each point existed in the channel actually. But there is a certain relationship between the flow speed at each position in the channel and the flow rate as shown in Figure 2(a). It can be seen that the probe alters the local flow speed actually as Figure 2(b) shows. It is the common problem for the current inserted-type flowmeter. In this paper, the flow rate can be controlled accurately by syringe pump and the flowmeter is calibrated with the flow rate.

**Figure 3:**
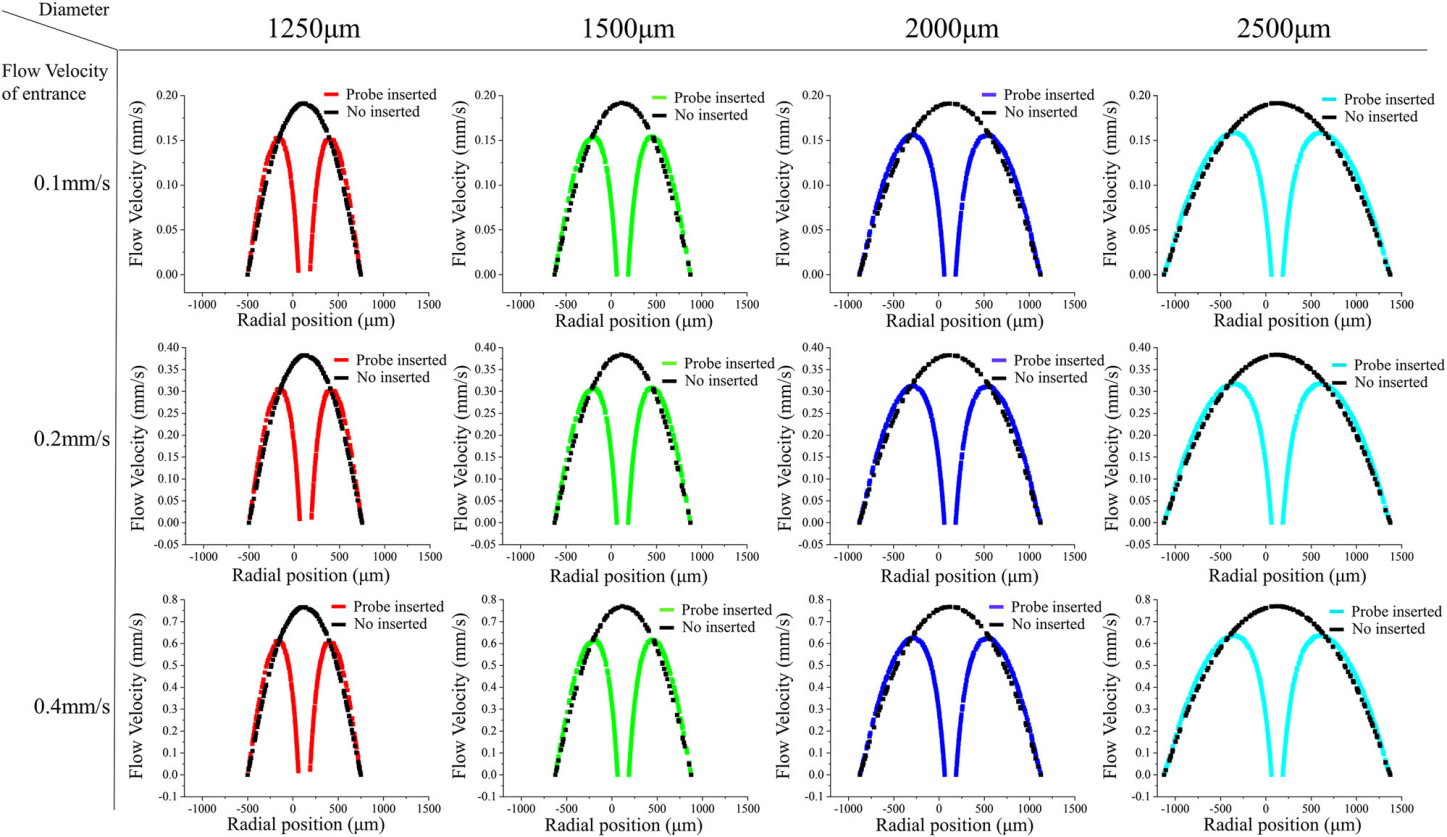
The effect of the insertion of the probe on actual flow velocity field in radial flow layer in channels with different diameter.

Besides, the effect of insertions of the probe on the actual flow velocity field in channels of various sizes is investigated. The flow velocity field of the radial flow field and the axis flow field has also been simulated, respectively as shown in Figure 3. When the flow velocity of entrance is 0.1 mm/s and the diameter of the channel is 1250 μm, the actual flow velocity in the center of the layer is higher than that of the sides and the flow velocity of the sides in the channel is close to 0 mm/s because of the effect of the side wall as the black curve shows. The maximum flow velocity can reach about 0.2 mm/s and is about twice higher than the flow velocity of entrance. When the probe with a length of 650 μm is inserted, the flow velocity field seems to be divided into two parts. The flow velocity can reach 0.15 mm/s respectively on both sides of the probe, which is reduced by 0.05 mm/s. As for the axis flow field, the probe can restrain the flow velocity and the flow velocity near the probe is close to zero in the axis flow layer. The insertion of the probe has less effect on the flow velocity field with the size of the channel increased.

Liquid flowing dominates the transportation of heat as microfluidics flow through the sensor probe; the convective flux boundary condition is applied:

(2) −k∇T⋅n=Q

The contact surface between the outer fluid and the external environment is set flux radiated outward as the heat convection. The energy radiated by the laser is equivalent to heat flux, which is coupled into the fiber microcavity through the core of SMF. The heat flux was set that can heat the silver nanoparticles solution in the microcavity to the same temperature as the 980 nm pump laser operating. Heat flux was used to heat the silver nanoparticles in microcavity. The solution in the cavity was heated continuously. As the fluid flows over the sensing probe, the heat generated by the SNS is taken away until temperature equilibrium is fixed under different velocity. Fabry–Pérot interference would occur when coherent light propagates through fiber microcavity. The interference dips will shift due to the different RI of the solutions at different temperatures caused by the change of the external flow velocity. Thus, the flow velocity can be measured by demodulating the shifting of the interference dips.

### 2.2 Characterization of probe of the flowmeter

**Figure 4:**
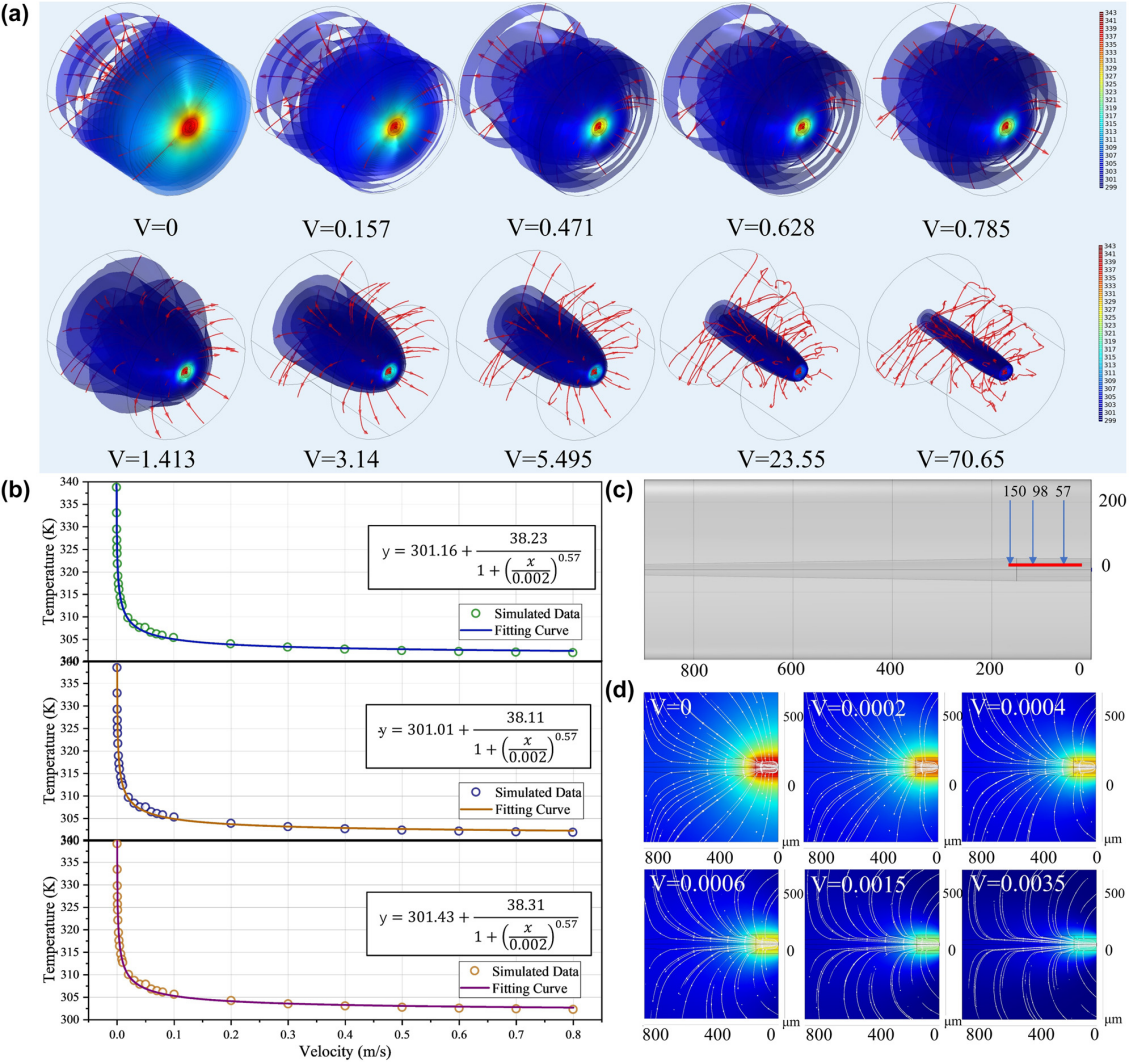
Simulation of the microfluidics flowmeter. (a) The FEA simulation of isothermal surface under different flow velocity. (b) Temperature variation and fitting curve at three point of the central axis under different flow velocity. (c) Sample point. (d) Temperature field section and surface streamline under different flow velocity.

FEA was used to simulate the performance of the flowmeter as shown in Figure 4. The temperature field distribution is solved when the sensor probe reached a thermal equilibrium under different flow rates. The isothermal surface and streamline are also depicted in a large range from 0 to 70.65 μL/s shown in Figure 4(a). It can be found that a tiny change in flow rate can lead to a large temperature change when the flow rate is low in the range of 0–0.785 μL/s. And as the flow rate increased, a change in flow velocity at the same scale will lead to a smaller temperature change relatively as show in Figure 4(a). To study the relationship between flow rate and steady-state temperature quantitatively, the temperature curve at 57 μm, 98 μm and 150 μm in the central axis under different flow rates are recorded, and fitting curves are shown in Figure 4(b) respectively, which is corresponding to the three points as shown in Figure 4(c). It can be also seen that the points in center axis have almost same sensitivities to the flow rate for the three fitting curves are nearly same to each other. The three R-squares (R2) are 99.98%, 99.98% and 99.98%, which indicate that the fitting curves match well with the experiment data, respectively. The simulation results for a point on the central axis of the flow rate are averaged. The relationship between the internal temperature of the flowmeter and the liquid flow velocity can be obtained as follows:

(3) $$T=339.5+\frac{38.2}{1+{\left(\frac{v}{0.002}\right)}^{0.57}}$$

Section temperature field distribution of the flowmeter probe is simulated under different flow velocity as shown in Figure 4(d). The streamline in the section slows with the increase of flow velocity. The temperature changes significantly as the flowrate is less than 0.785 μL/s and when the flowrate increased gradually, temperature at thermal balance changes smaller.

**Figure 5:**
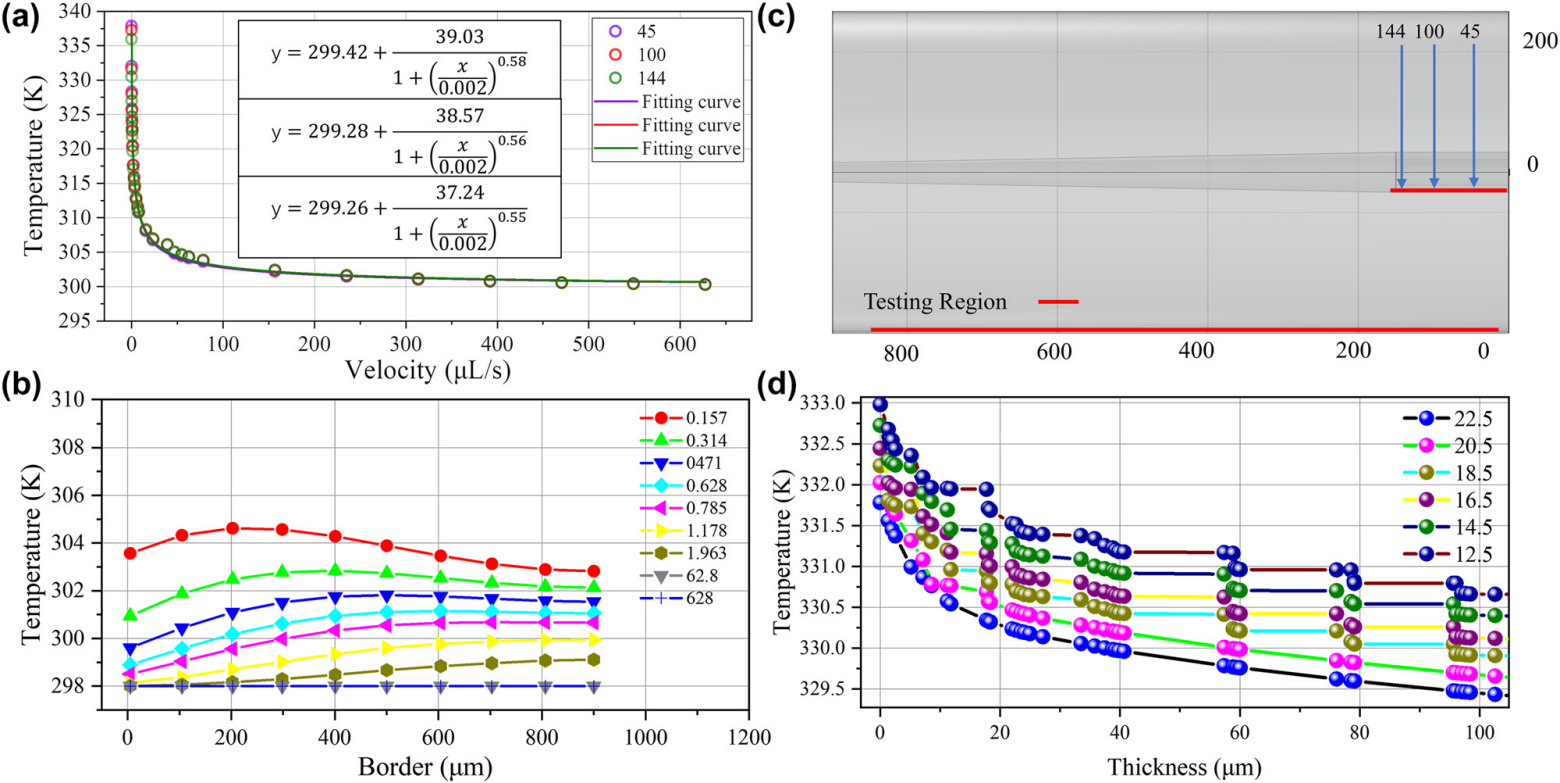
Simulation results of the flowmeter: (a) Temperature variation and fitting curve at three points of the central axis of side wall under different flow rate. (b) Temperature variation along the boundary of fluid and air under different flow rate. (c) Sample point. (d) Steady-state temperature along the central axis versus thickness of sidewall (superficial area) under flow rate of 0.314 μL/s.

The overall data and fitting results are shown in Figure 5(a) and it can be seen that three curves almost overlap with each other. At different flow rates, the side wall temperature is a little lower than the temperature in the cavity and the overall fitting curve is similar to the temperature curve of silver nanoparticles in the cavity. That is to say, the flowmeter probe has a good thermal conductivity, which will improve the performance of the flowmeter undoubtedly. The boundary temperature curve between the fluid and the outside air is shown in Figure 5(c). It is worth noting that when the measurement range is more than 600 μm, the temperature change is not obvious, so the microfiber microcavity flowmeter can monitor the flow rate within the 600 μm range, which has an essential guiding significance as assembling distributed probe. To further optimize the performance of the micro flowmeter, the steady state temperature curve is simulated under 0.314 μL/s fluid rate with different thickness of the sidewall from 12.5 μm to 22.5 μm. We found that when the thickness of the sidewall decreases, the temperature of thermal balance increased, as shown in Figure 5(d). As the thermal conductivity of silica is larger than that of water, the influence of thermal conductivity can be excluded. We believe that the reduction of the surface area of the fiber microcavity matters. As the thickness of the sidewall decreased, the surface in contact with the external fluid decreased, which is not conducive to the heat dissipation. Therefore, we can increase the surface area to improve the flow rate sensitivity and DL.

## 3 Results and discussion

**Figure 6:**
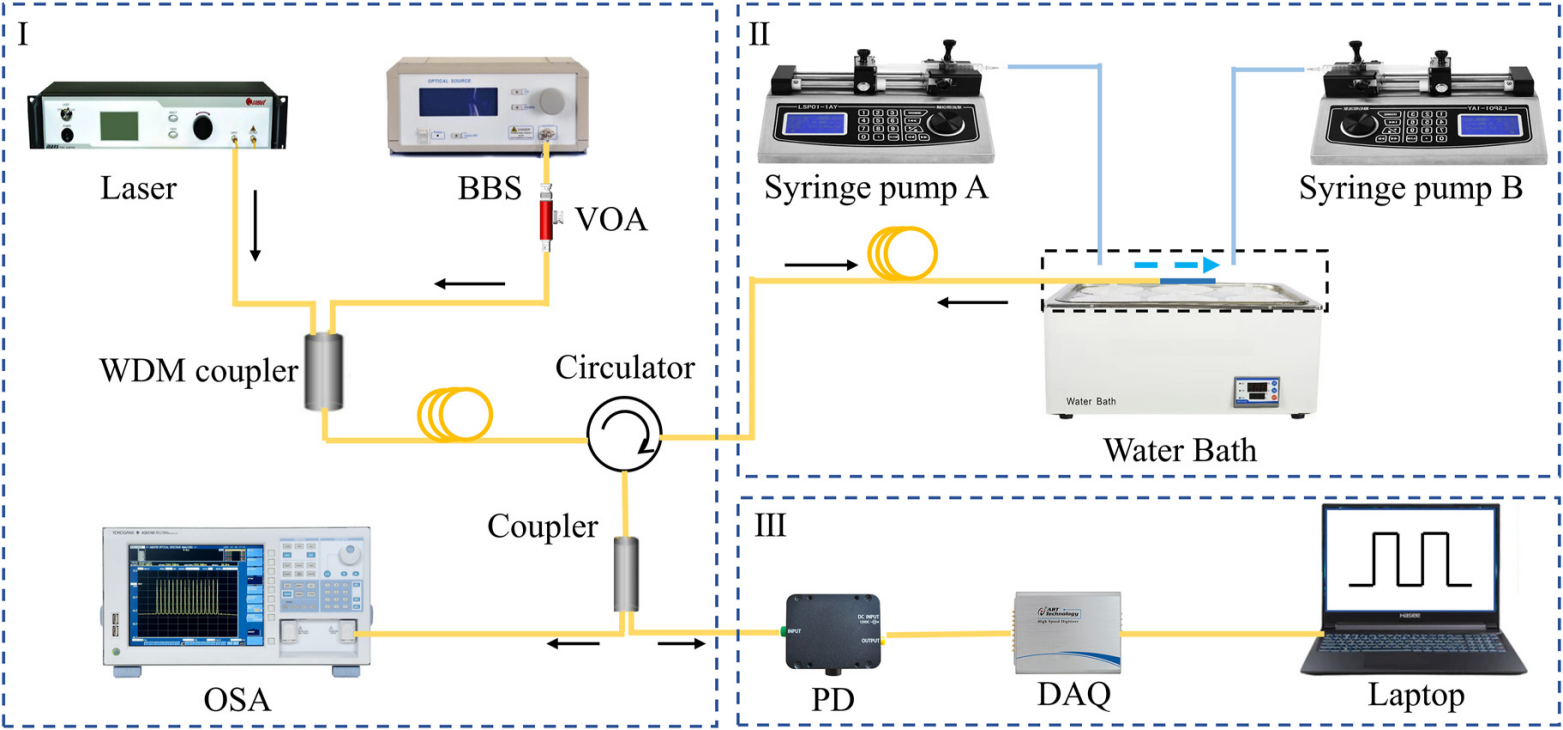
Schematic of the fluidics flow rate measuring system. BBS, broadband light source; VOA, variable optical attenuator; WDM, wavelength division multiplexer; PD, photoelectric detector; DAQ, high-speed data acquisition card.

Figure 6 shows measuring and demodulating system for the flowmeter. A 980 nm pump laser with a tunable power is used to heat the SNS in microcavity. The light from the pump laser and the light from broadband light source (BBS) travelling through the circulator are coupled into the WDM and connect to C1 of the circulator. A VOA is introduced to reduce the light intensity energy generated by the BBS light source, avoiding the incidental heating of the silver nanoparticles. The light propagates via C2 and arrived at the sensor structure. The reflected light comes out of the C3 circulator and is split into two beams through the coupler 2 (50:50). One beam of light is collected by a spectrometer and the other is converted into electrical signals by a photodetector and collected by a high-speed acquisition card, the response time is measured by the self-designed program. Figure 6 also shows the flowrate monitoring part. The probe is packaged into a channel with diameter of 2000 μm, and two single channel syringe pumps (Rongbai, LSP01-1AY) are employed to control the flowrate. The whole sensing structure is put into the water bath and the temperature crosstalk is also studied.

**Figure 7:**
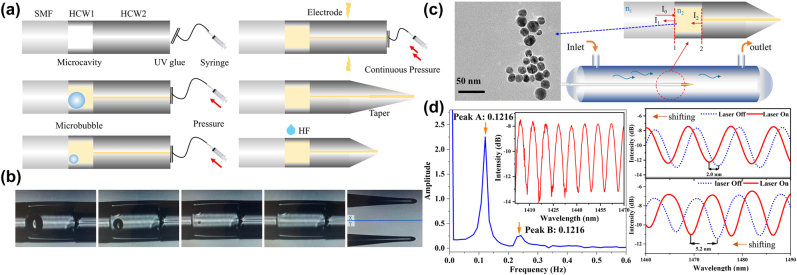
Fabrication of the flowmeter: (a) Fabrication process of the fiber-tip flowmeter probe. (b) Images of the fabrication process. (c) Schematic diagram of the fiber-tip flowmeter probe; the inset is the TEM images of silver nanoparticles solution. (d) FFT and reflection spectra of sensor probe; reflection spectra before and after immersed in liquid with the pump laser.

Figure 7(a) shows the fabrication process of the microfluidics flowmeter. Based on our previous work [40]. Single mode fiber (SMF) is spliced with a piece of hollow optical waveguide (HCW) −1 with an outer diameter of 125 μm and inner hollow core diameter of 80 μm. The spliced HCW-1 is cleaved by the three-dimensional high precision cutting platform. HCW-2 with an outer diameter of 125 μm and inner diameter of 10 μm is filled with a piece of SNS because of the capillarity effect. The other end of HCW-2 is attached to a high-pressure syringe and sealed with UV glue. The HCW-2 is spliced with the cleaved SMF-HCW-1 structure by proper arc discharge and a little fraction of silver nanoparticles solution is ionized near the discharge position. The SNS in HCW-2 was continuously pushed into the closed microcavity of HCW-1 by using a high-pressure syringe, and the air in the cavity was gradually emptied. Continue high pressurizing until air is fully expelled, and no air enters the microcavity any more after the pressurizing device is removed. It can also be seen that each fiber fusion point is relatively flat and does not have a large impact on the reflection spectrum as shown in Figure 7(b). And then, arc discharge is used to taper the position about 500 μm away from the microcavity and the SNS is sealed. This process also ensures that there is no light bouncing back and affecting the spectrum. A structure diagram of the microfluidics flowmeter probe is shown in Figure 7(c). The fiber end facets of SMF and HCW-2 both act as partially reflective mirrors and HCW-1 is used as a resonant cavity. And then the FP interference cavity is constructed by M1 and M2. The measured length of *L* is 130 μm for the FP cavities. We can see that when a light beam I_0_ travels from the lead-in fiber, it is reflected by reflectors M_1_ and M_2_, respectively. The dip wavelength of the reflection spectrum caused by the liquid FP and the free spectral range (FSR) of microcavity can be expressed as [41]:

(4) $$\text{FSR}=\lambda_{m+1}-\lambda_{m}=\frac{\lambda_{m}\lambda_{m+1}}{2h\sqrt{n_{1}^{2}-n_{0}^{2}}}$$

(5) $$\lambda_{m}=\frac{4n_{\text{liquid}}L}{2m_{\text{th}}+1}\;m=0,1,2,\ldots$$

Where *λ*_*m*_ is the dip wavelength of the *m*_th_ interference fringe, *n*_liquid_ is the RI of the liquid in micro cavity. In addition, Figure 7(d) also proves that the reliability of our taper packaging process due to its good FP interference spectrum. The reflection spectrum mainly contains quasi-sinusoidal pattern coarse fringes from the liquid FP cavity I. Due to the transmission loss of the light power in the liquid regions and reflection loss on the reflectors, the intensities of modes in cavity II are weak enough and only produce the slight fluctuations on the interferometric spectrum. The corresponding spatial frequency spectrum obtained by fast Fourier transform (FFT) is shown in Figure 7(d). We think that the small peak in the interference spectrum corresponds to the Peak 2 in the Fourier transform caused by the extra FP cavity II. Part of the light propagates through cavity | and arrived at cavity II, fixed by the end of taper, which will not affect the flow rate monitoring. Besides, it can also be proved that in Figure 7(d). The concentration of the silver nanoparticle solution is 100 ppm, and the size of the silver nanoparticle is 15 ±  5 nm. The SNS is the dispersion solution, which does not guarantee that silver nanoparticles are uniformly distributed in the microcavity absolutely. The temperature may be higher in the places with more silver nano-ions accumulated. The optical fiber microcavity is tiny enough when operated in the channel with a diameter of 2000 μm. The heat transfer is so fast in the cavity that the temperature distribution can be considered uniformly in such a small microcavity. The whole fiber microcavity can be considered as a heat source. The filling of silver nanoparticles mainly leads to the increase of the optical field transmission loss in the cavity, and there are no other interference peaks, which indicates that the size of silver nanoparticles is suitable, and does not affect the optical interference. The heat dissipation of microfluidics flow velocity sensor is [42]:

(6) $$Q=\left(A+Bv^{n}\right)\Delta T$$

Where Δ*T* is the temperature change; *A*, *B* and *n* are constants related to the microfluidic properties, the channel environment, respectively, and the microfluidic channel, roughness, and other factors. The heat loss of the optical fiber sensing probe increases as the flowrate in increases, and the temperature of thermal balance decreases causing the effective RI changed. The dip of interference wavelength shifted by an amount Δ*λ*, the sensitivity is estimated as:

(7) $$\Delta \lambda =\frac{4n_{\text{liquid}}L}{2m_{\text{th}}+1}+\frac{L\text{d}n_{\text{liquid}}}{\text{d}T}\cdot \Delta T$$

It can be seen that the interference dip moves toward the short wavelength as the temperature increase. SNS acts as an intermediate medium and is heated continuously. The RI of the solutions changes due to its strong optic-thermo effect. Then the change of FP interference spectrum presents as the movement of interference dip as shown in Figure 7(d), the dips of which shifts a length of 5.2 nm in the air and 2.0 nm in liquid under input power of 75 mW, which is larger than that of FBG as shown in Table 1.

**Table 1:** Comparison of different hot wire liquid flow sensor technologies.

Sensor structure	△λ/Heat power	Size	Sensitivity	Ref
Micro FBG with Co^2+^ doped	0.54 nm/500 mW	30 mm*125 μm	0.31 nm/(μL/s)	[37]
Co^2+^ doped fiber FPI	0.54 nm/400 mW	Over 500 μm*125 μm	70 pm/(μL/s)	[42]
Microfiber coupler	14 nm/200 mW	About 1.2 mm*5 mm	2.183 nm/(μL/s)	[36]
Microcavity	7.6 nm/75 mW	300 μm*130 μm	1.5 nm/(μL/s)	This work

**Figure 8:**
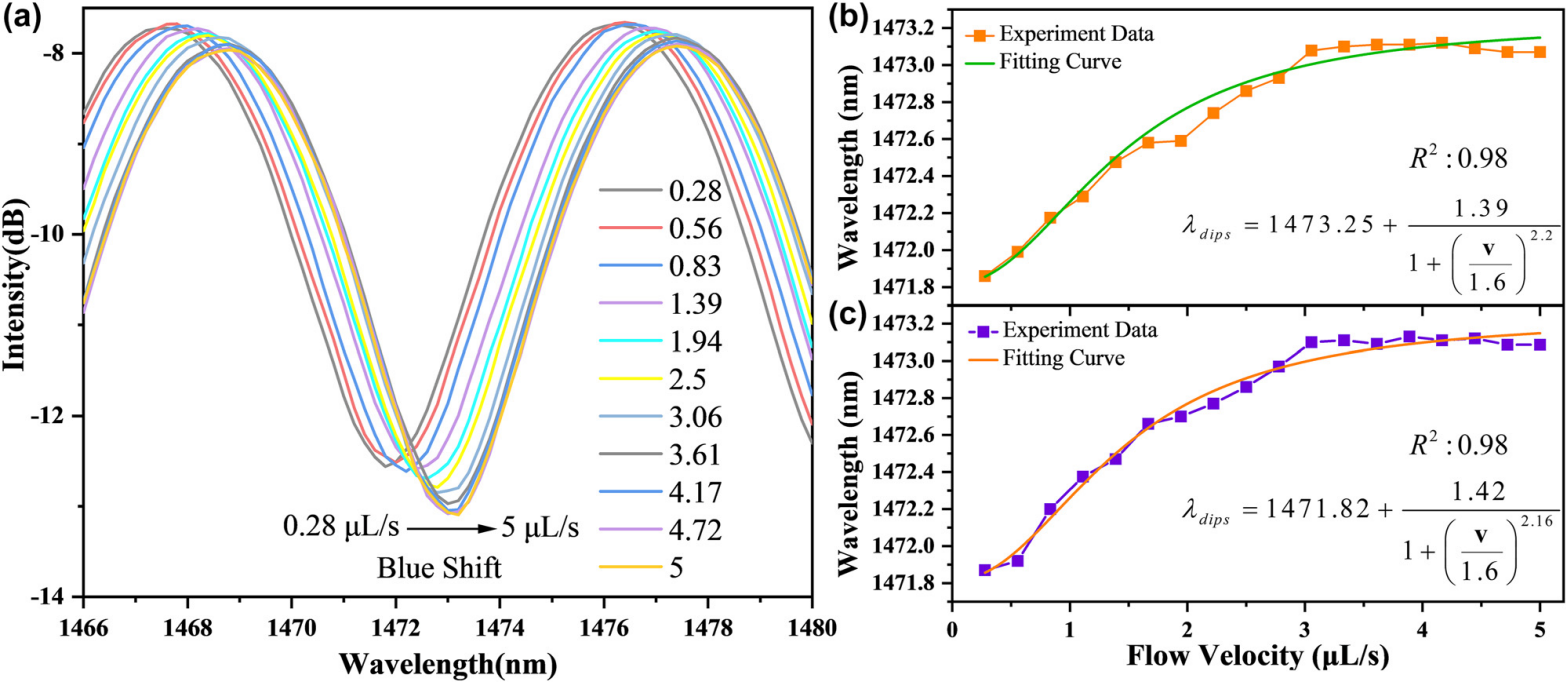
Demodulation of spectra: (a) The transmission spectra of microfluidic flowmeter under different flow velocity in positive direction. (b) Fitting of the dip shifting at 1473.25 nm in positive direction. (c) Fitting of the dip shifting at 1471.82 nm in opposite direction.

The flowmeter is fixed in the channel and a high-precision five-position displacement platform is used with a resolution of 5 μm. The cylindrical channel has a diameter of 2000 μm and is linked to a syringe pump. The detection range will increase with the laser power [42], which is also suitable for the fluid flowmeter sensing structure. Therefore, the max output power of the power-tunable laser is stabilized with 500 mW for heating to obtain a larger detection range. The microfluidics is injected with different speeds by syringe pumps. The microfluidics is injected with different speeds by syringe pumps. The data is recorded until the spectrum is stabilized when the thermal balance is established between the heat taken away by the fluid and the heat generated by SNS. It can be found in Figure 8(a) that, the interference dips shift to the short wavelength direction with the increase of flow rate, which is consistent with the Eq. (7). As the flow rate increases, the temperature of the thermal balance decreases, and the interference dips shift to longer wavelength. Afterwards, we recorded the shift of the interference dips and performed a fitting to it as shown in Figure 8(b), the fitting curve of which can be consistent well with the simulated results.

**Figure 9:**
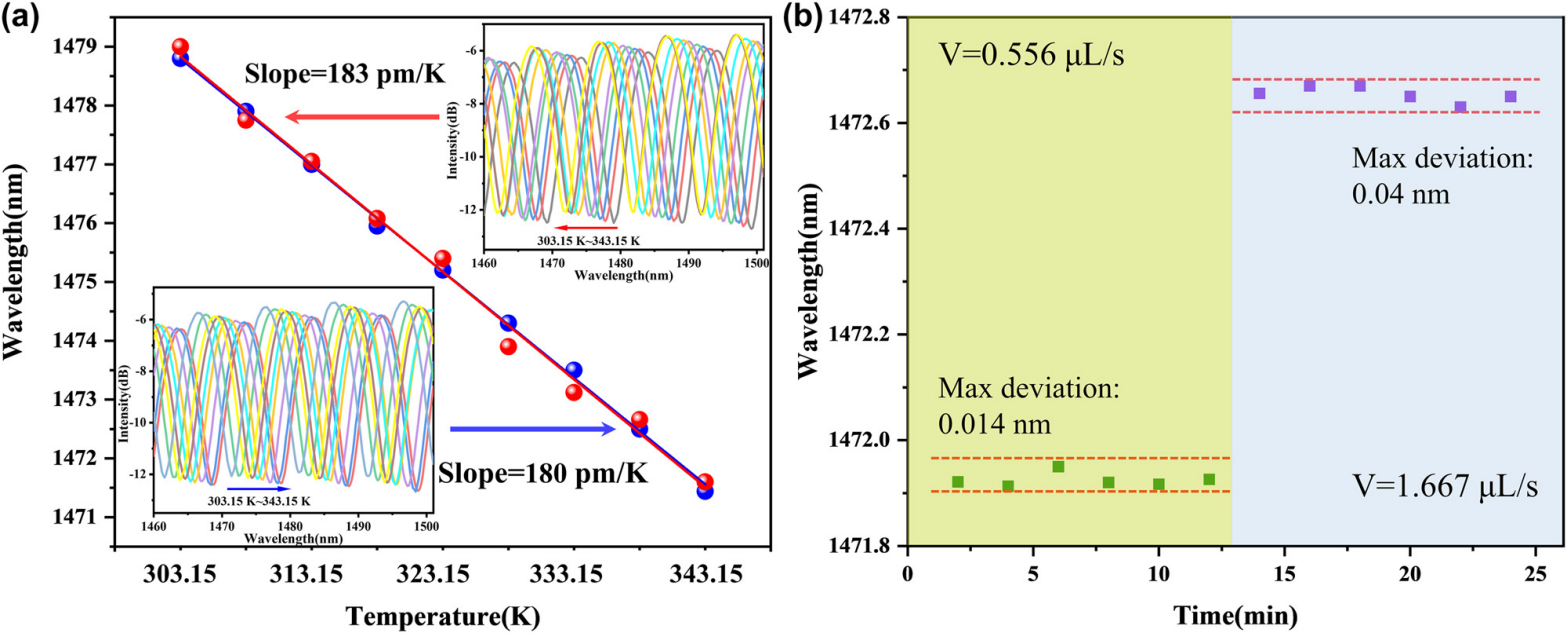
Temperature response and stability test: (a) Temperature characteristic of the sensor probe. (b) Stability test under flow velocity of 0.556 μL/s and 1.667 μL/s.

In order to verify the performance of the flowmeter at different temperatures, temperature tests are performed that similar sensor probe is immersed in a water bath. The temperature is increased from 313.15 to 343.15 K with a step of 10 K. The transmission dips shift slightly to shorter wavelength as temperature rises seen in Figure 9(a) and the experimental temperature sensitivity is 183 pm/K. The sensitivity can also reach 180 pm/K when temperature decreasing. Therefore, the flow velocity sensing device has a good temperature response. We use the water bath heating in Figure 4 to further study the flow velocity characteristics of the flowmeter at different temperature. The flow rate is monitored from 0 to 3 μL/s at 293.15 K, 313.15 K, and 333.15 K, and the results are consistent with the Ref [42]. We believe that the SNS heated by the CW laser is based on the ambient temperature, so it does not affect the difference of temperature between the solutions in microcavity. Therefore, the flowmeter can also be used for the measuring the flow rate of fluid at different temperature. Stability test has also been carried out. The flowmeter operated under flow rate of 0.556 μL/s and 1.667 μL/s. The reflection spectrum was recorded every 2 min in 12 min, respectively, and the results as shown in Figure 9(b).

To further analyse the response performance of the flowmeter. A tunable laser (OPEAKTech, TLS-3000), photoelectric detector (Thorlabs, DET08CFC) and high-speed acquisition card (Smacq, USB-1000) were used to record the sensor response time continually and the data were transferred to computers. The rapid conversions of the flow rate in channel between 0.556 μL/s and 1.667 μL/s can be achieved, respectively. When the flow rate changed rapidly, the voltage variation occurred, and the voltage could return to the initial value (around the average voltage value) after each cycle. The rise time and recover time of the flowmeter are 32 s and 37 s, which is consistent with the characteristic of the hot-wire flowmeter. Table 1 compares the performance of different “hot wire” technologies. In terms of flow rate sensitivity, the proposed fiber-tip fluid flowmeter with micro cavity is higher than most other hot-wire flowmeters. In addition, the flowmeter has the advantages of high mechanical strength, probe-type structure, ultra-compactness.

## 4 Conclusions

In conclusion, we demonstrated a novel fiber-tip flowmeter based on optofluidic microcavity filled with SNS. The SNS in the microcavity are used as the heat source and the heat is carried away by the external fluid flowing over the probe. This principle was applied to the measurement of flow rate firstly as far as we know. The flowmeter has a high sensitivity due to the excellent optic-thermo performance of the SNS. The max sensitivity can reach 1.5 nm/(μL/s) with a DL of 0.08 μL/s. And the size of the flowmeter is only 2 mm, which has great application potentials in hematology, oil exploration, ocean dynamics, and drug research, due to its ultracompact structure, high sensitivity, and stability.
